# Supplementary material for: The Drosophila miR-959–962 Cluster Members Repress Toll Signaling to Regulate Antibacterial Defense during Bacterial Infection
Source: Int J Mol Sci. 2021 Jan 17;22(2):886. doi: 10.3390/ijms22020886 (PMC7831006; doi:10.3390/ijms22020886)
Supplement: Supplementary file 1 [file ijms-22-00886-s001.zip › ijms-1053482-supplementary/Supplementary Files/Table S1.docx]

**Table S1. Primers used for quantitative RT-PCR:**

| Name | Primer sequence |
| --- | --- |
| rp49-F | 5’- GACGCTTCAAGGGACAGTATCTG -3’ |
| rp49-R | 5’- AAACGCGGTTCTGCATGAG -3’ |
| Drs-F | 5’- CGTGAGAACCTTTTCCAATATGATG -3’ |
| Drs-R | 5’- TCCCAGGACCACCAGCAT -3’ |
| tube-F | 5’- AACTCTCGACCAAATCACGCTCCA -3’ |
| tube-R | 5’- AAGGTCTCCCTGCTGCCTTTACTT -3’ |
| dl-F | 5’- GAGCAGAAGGGTCGATTCAC -3’ |
| dl-R | 5’- TCGGACATGGCCTTCTTATC -3’ |
| Toll-F | 5’- AGAGCGACGTATAGGACT -3’ |
| Toll-R | 5’- ACCTATAAGAGGGCGACT -3’ |
| U6-F | 5’-CTTCGGCAGAACATATACTAA -3’ |
| U6-R | 5’-ATTTTGCGTGTCATCCTT -3’ |
| miR-959-qF | 5’- GCGTTGTCATCGGGGGTAT -3’ |
| miR-960-qF | 5’- GCGTGAGTATTCCAGATTG -3’ |
| miR-961-qF | 5’- GCGTTCGTTTTCTGGCAAT -3’ |
| miR-962-qF | 5’- GCGATAAGGTAGAGAAATT -3’ |
| miRNA-qR | 5’- CAGTGCAGGGTCCGAGGTAT -3’ |
| miR-959-RT | 5’- GTCGTATCCAGTGCAGGGTCCGAGGTATTCGCACTGGATACGAC  TTCATA -3’ |
| miR-960-RT | 5’- GTCGTATCCAGTGCAGGGTCCGAGGTATTCGCACTGGATACGAC  GCTATG -3’ |
| miR-961-RT | 5’- GTCGTATCCAGTGCAGGGTCCGAGGTATTCGCACTGGATACGAC  CTTTTC -3’ |
| miR-962-RT | 5’- GTCGTATCCAGTGCAGGGTCCGAGGTATTCGCACTGGATACGAC  GACAGC -3’ |
