# Supplementary material for: The Drosophila miR-959–962 Cluster Members Repress Toll Signaling to Regulate Antibacterial Defense during Bacterial Infection
Source: Int J Mol Sci. 2021 Jan 17;22(2):886. doi: 10.3390/ijms22020886 (PMC7831006; doi:10.3390/ijms22020886)
Supplement: Supplementary file 1 [file ijms-22-00886-s001.zip › ijms-1053482-supplementary/Supplementary Files/Table S2.docx]

**Table S2. Primers used for transgene vector construction:**

| Name | Primer sequence |
| --- | --- |
| miR-959-F | 5’- GAATTCGCAAAATGTGCCAGC -3’ |
| miR-959-R | 5’- CTCGAGTGGGCGGTACGATAA -3’ |
| miR-960-F | 5’- GAATTCCTGCAATGATTATCGTACC -3’ |
| miR-960-R | 5’- CTCGAGGCTCACTACGGATTCTATTA -3’ |
| miR-961-F | 5’- GAATTCCTAATAGAATCCGTAGTGAG -3’ |
| miR-961-R | 5’- CTCGAGATTCAAGTCGAGTCCAA -3’ |
| miR-962-F | 5’- GAATTCAATTCAACTACTTCGAT -3’ |
| miR-962-R | 5’- CTCGAGGTGTATTGTCTGGTCC -3’ |
| tube 3’UTR-F | 5’- GAATTCTAAAAGCACCGACCAT -3’ |
| tube 3’UTR-R | 5’- CTCGAGTTTTATTTGTATTTAG -3’ |
| dl 3’UTR-F | 5’- GAATTCATTTGTTGATTTGATTGT -3’ |
| dl 3’UTR-R | 5’- CTCGAGGTTTTATTAAGCTAC -3’ |
| Toll 3’UTR-F | 5’- GAATTCGACTGGGAGAAGGCGGAGC -3’ |
| Toll 3’UTR-R | 5’- CTCGAGTATTTGCGTTGTATGTGTTTTT -3’ |
