# Supplementary figures and images for: The Drosophila miR-959–962 Cluster Members Repress Toll Signaling to Regulate Antibacterial Defense during Bacterial Infection
Source: Int J Mol Sci. 2021 Jan 17;22(2):886. doi: 10.3390/ijms22020886 (PMC7831006; doi:10.3390/ijms22020886)

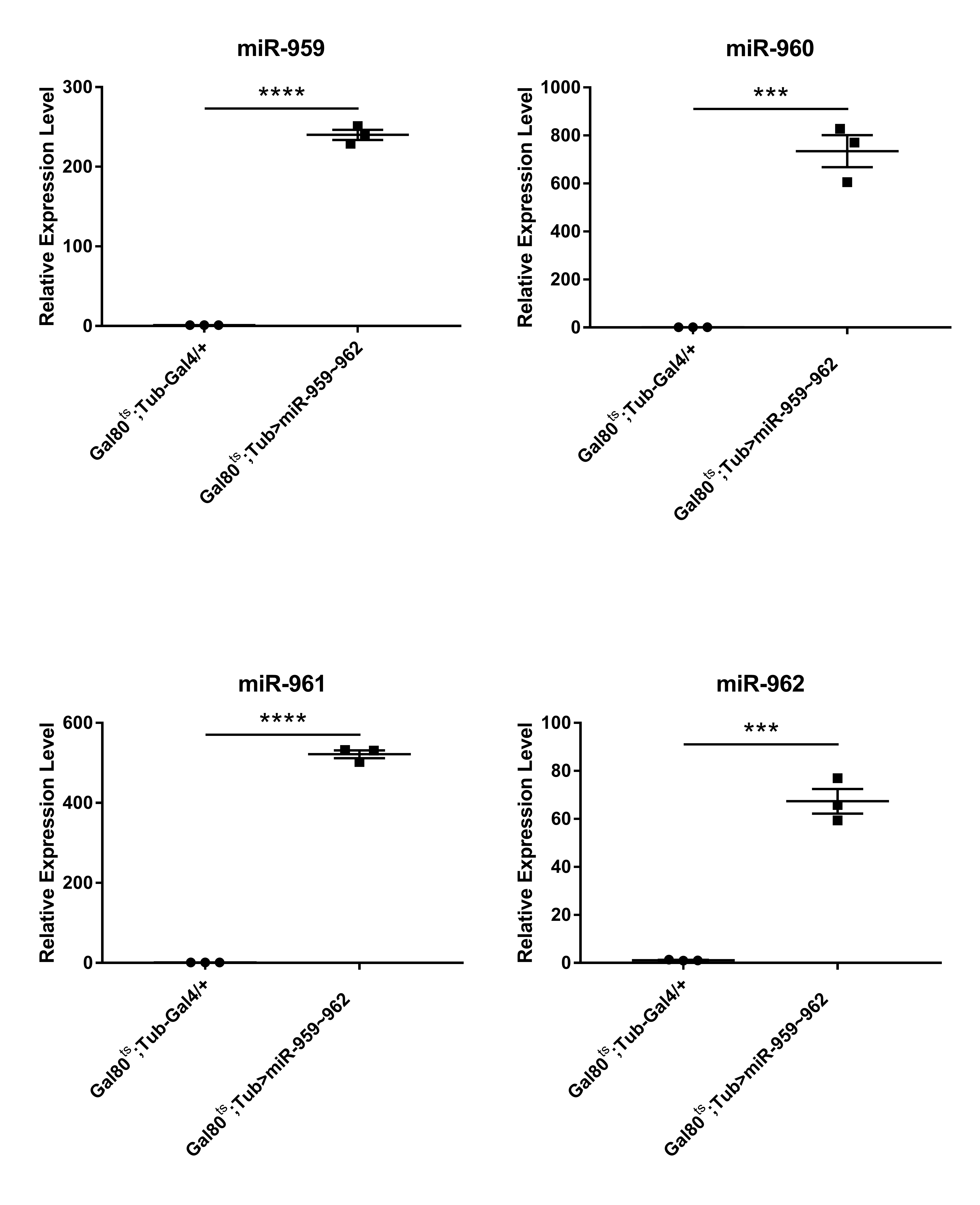

Supplement: Supplementary file 1 [file ijms-22-00886-s001.zip › ijms-1053482-supplementary/Supplementary Files/Figure S1.tif]

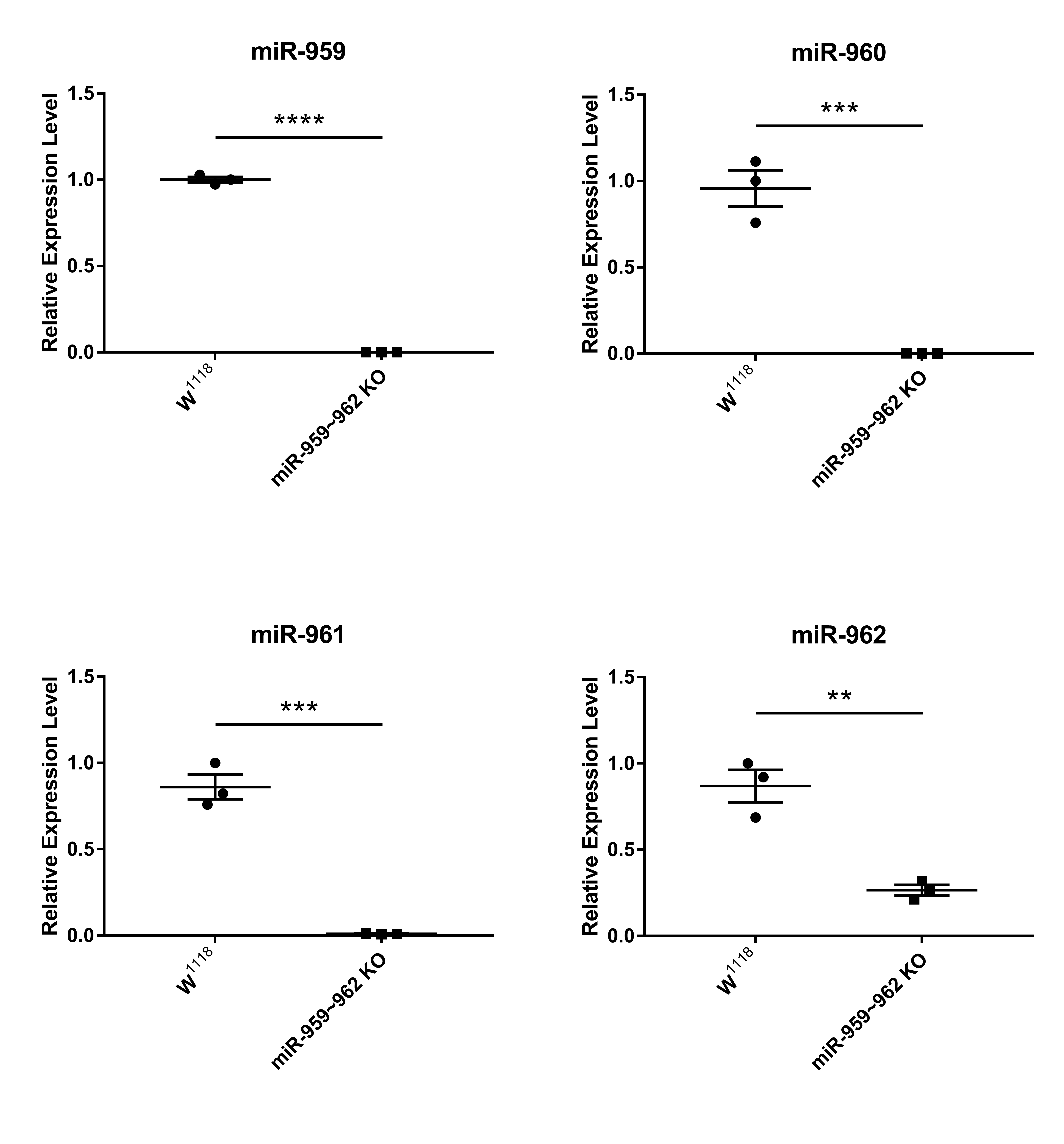

Supplement: Supplementary file 1 [file ijms-22-00886-s001.zip › ijms-1053482-supplementary/Supplementary Files/Figure S2.tif]

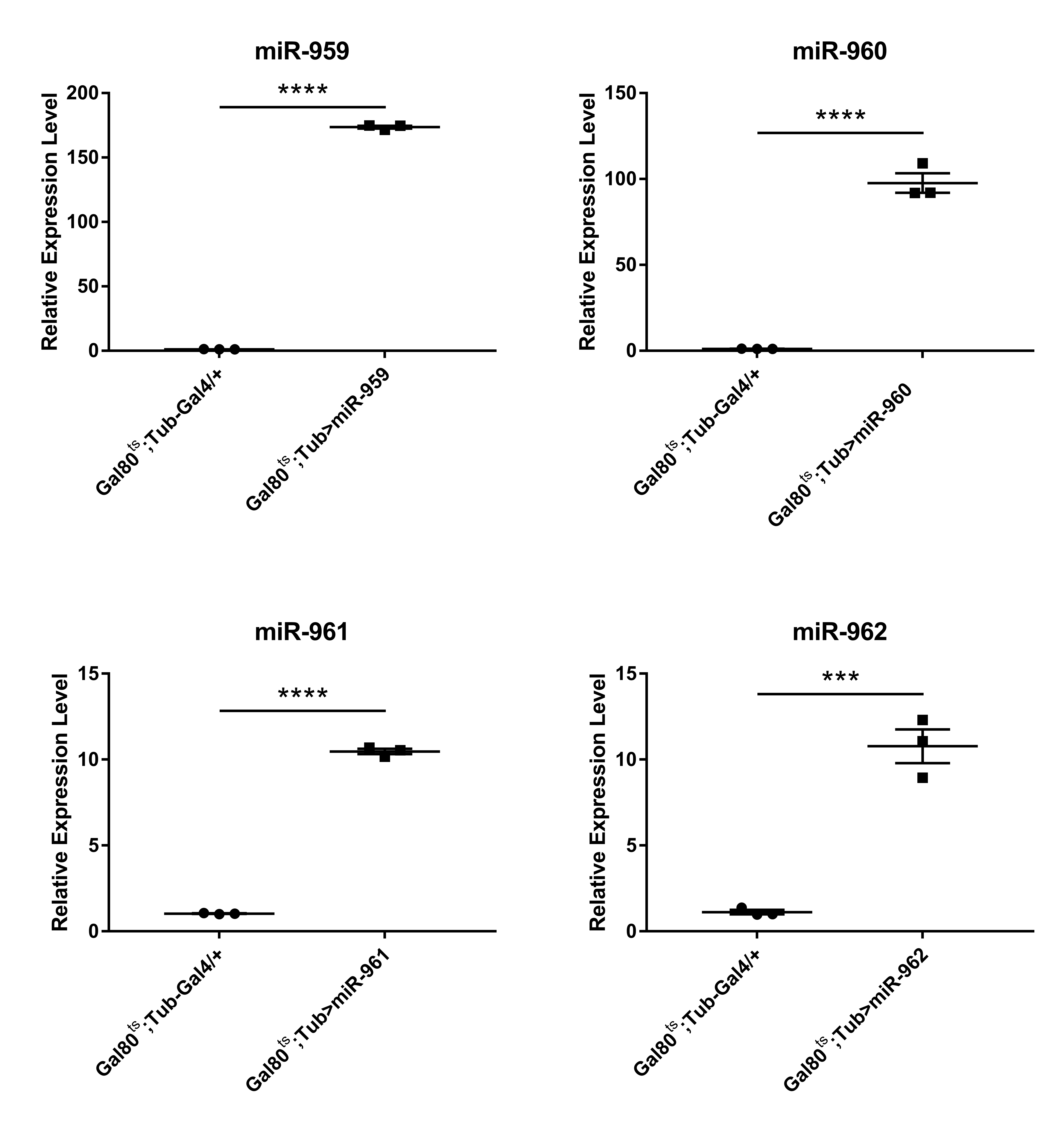

Supplement: Supplementary file 1 [file ijms-22-00886-s001.zip › ijms-1053482-supplementary/Supplementary Files/Figure S3.tif]
